# Peroxisome Proliferator Activator Receptor Gamma Coactivator-1α Overexpression in Amyotrophic Lateral Sclerosis: A Tale of Two Transgenics

**DOI:** 10.3390/biom10050760

**Published:** 2020-05-13

**Authors:** Merina Varghese, Wei Zhao, Kyle J. Trageser, Giulio M. Pasinetti

**Affiliations:** 1 Department of Neurology, Icahn School of Medicine at Mount Sinai, New York, NY 10029, USA; merina.varghese@mssm.edu (M.V.); wei.zhao@mssm.edu (W.Z.); kyle.trageser@mssm.edu (K.J.T.); 2 Geriatric Research, Education and Clinical Center, James J. Peters Veterans Affairs Medical Center, Bronx, New York, NY 10468, USA

**Keywords:** mitochondrial biogenesis, motor function, neurodegeneration, tissue-specific expression

## Abstract

Amyotrophic lateral sclerosis (ALS) is a progressive neurodegenerative disorder manifesting with upper and lower neuron loss, leading to impairments in voluntary muscle function and atrophy. Mitochondrial dysfunction in metabolism and morphology have been implicated in the pathogenesis of ALS, including atypical oxidative metabolism, reduced mitochondrial respiration in muscle, and protein aggregates in the mitochondrial outer membrane. Peroxisome proliferator-activated receptor γ coactivator-1α (PGC-1α) plays an essential role in the regulation of mitochondrial biogenesis, the process by which existing mitochondria grow and divide. PGC-1α has been previously reported to be downregulated in the spinal cord of individuals with ALS. Towards targeting PGC-1α as a therapeutic mechanism, we have previously reported improved motor function and survival in the SOD1^G93A^ mouse model of ALS by neuron-specific over-expression of PGC-1α under a neuron-specific enolase (NSE) promoter. As pharmacological intervention targeting PGC-1α would result in whole-body upregulation of this transcriptional co-activator, in the current study we investigated whether global expression of PGC-1α is beneficial in a SOD1^G93A^ mouse model, by generating transgenic mice with PGC-1α transgene expression driven by an actin promoter. Actin-PGC-1α expression levels were assayed and confirmed in spinal cord, brain, muscle, liver, kidney, and spleen. To determine the therapeutic effects of global expression of PGC-1α, wild-type, actin-PGC-1α, SOD1^G93A^, and actin-PGC-1α/SOD1^G93A^ animals were monitored for weight loss, motor performance by accelerating rotarod test, and survival. Overexpression of actin-PGC-1α did not confer significant improvement in these assessed outcomes. A potential explanation for this difference is that the actin promoter may not induce levels of PGC-1α relevant to disease pathophysiology in the cells that are specifically relevant to the pathogenesis of ALS. This evidence strongly supports future therapeutic approaches that target PGC-1α primarily in neurons.

## 1. Introduction

Amyotrophic lateral sclerosis (ALS), also known as Lou Gehrig’s disease, is a neurological disorder affecting motor neurons and leading to progressive loss of voluntary muscle function, paralysis, and ultimately death. ALS can be either inherited or sporadic; whereas 10% of cases are familial ALS (fALS) with inherited mutations in genes including *TDP-43*, *FUS*, *C9ORF72*, and *SOD1*, 90% are classified as sporadic ALS (sALS), for the majority of which the cause is unknown [1].

The etiopathogenesis of ALS is incompletely understood. However, it is believed that ALS is a multifactorial disease with deficits in various facets of the normative function of the cell, including oxidative stress and aberrant mitochondrial function [2]. Mitochondrial bioenergetics are increased in the neocortex of patients with fALS as compared to controls [3,4]. In human Cu/Zn superoxide dismutase 1 (SOD1) mouse models of ALS, ALS-associated variants of SOD1 protein were abnormally imported into spinal cord mitochondria [5,6]. The SOD1^G93A^ mutant, a commonly studied model of fALS, causes decreased mitochondrial respiration [6,7] and increased fragmentation of the organelle [8] compared to wild-type controls, leading to loss of neurons in the anterior horn of the spinal cord [9]. In vitro studies implicate similar mitochondrial functional and structural changes in fibroblasts expressing mutant *TDP-43* and *C9ORF72* [10]. Mitochondrial functional changes have also been reported in sALS, including reduced mitochondrial respiration in skeletal muscle [11] and fibroblasts [12], increased bioenergetic function in fibroblasts [13], but no alteration in mitochondrial respiration in the spinal cord [4]. Reports of changes in the complexity of the mitochondrial network vary, with either loss of age-related complexity [12] or no changes [14] reported. Mitochondrial abnormalities may represent a disease mechanism shared by both sALS and fALS [15].

The altered mitochondrial structure and function observed in ALS may result from the dysfunction of various mechanisms, which under normal conditions act to maintain proper homeostasis of the organelle. Affected homeostatic mechanisms in ALS include altered axonal transport, fission/fusion, mitophagy, and biogenesis of mitochondria. Mitochondrial biogenesis, the process by which mitochondria grow and replicate, is regulated by the transcription co-activator peroxisome proliferator-activated receptor gamma, coactivator 1 alpha (PPARGC1A or PGC-1α) [16]. Underscoring the relevancy of PGC-1α to the pathogenesis of ALS, PGC-1α expression has been shown to be reduced in the muscles [17,18], motor cortex, and spinal cord motor neurons of ALS patients^17^, as well as in muscle [17,19] and spinal cord of the SOD1^G93A^ mouse model of ALS [17].

We hypothesized that increasing mitochondrial mass through PGC-1α over-expression may lead to protection in ALS neurodegeneration. A previous study from our group found improved motor function and survival following neuronal overexpression of PGC-1α in the SOD1^G93A^ mouse model of ALS, driven by the neuron-specific enolase (NSE) promoter [20]. Therapeutic interventions often have pleiotropic, off-target effects on unintended cell types, and delivery to a specific cell type may not directly translate to clinical practice. Therefore, we investigated if beneficial effects could be achieved in the same mouse model used for our previous studies if PGC-1α is increased throughout the body. To better understand the effects of PGC-1α over-expression in the whole body, we tested the effects of the transgene expressed by the ubiquitous beta-actin promoter, which is active in most cells, including neurons, in the SOD1^G93A^ mice on body weight, motor function, and survival.

## 2. Materials and Methods

### 2.1. Generation of Actin-PGC-1α Mice and Crossing with SOD1^G93A^ Mice

**Figure 1:**
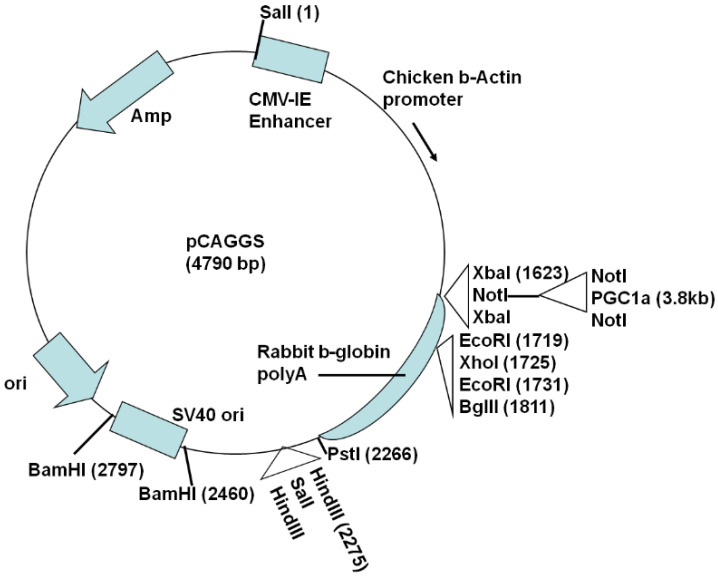
Plasmid construct for generation of actin-PGC-1α transgenic mice. A *Not*I site was inserted at the vector *Xba*I site and a *Sal*I site inserted at the vector *Hind*III site on the pCAGGS plasmid. Human peroxisome proliferator-activated receptor gamma, coactivator 1 alpha (PGC-1α) cDNA was inserted in the *Not*I site and *Sal*I digestion released the fragment containing the cytomegalovirus immediate-early (CMV-IE) enhancer, chicken β-actin promoter, and hPGC-1α that was used for microinjection.

For global PGC-1α transgene expression driven by the actin promoter, the plasmid vector pCAGGS ^20^ containing the cytomegalovirus immediate-early (CMV-IE) enhancer and chicken β-actin promoter was modified by inserting a *Not*I site at the vector *Xba*I site and a *Sal*I site at the vector *Hind*III site. Then, the 3.1 kb human PGC-1α (hPGC-1α) cDNA (NM_013261.2; obtained from OriGene Technologies, Inc., Rockville, MD, USA) was inserted in the *Not*I site (Figure 1), followed by *Sal*I digestion to release a ~6 kb fragment containing the CMV-IE enhancer, chicken β-actin promoter, and hPGC-1α. The fragment was gel purified and microinjected into one-cell mouse eggs as described previously [20]. Actin-PGC-1α founders were identified by PCR-based genotyping using the primers in Table 1. The generation of NSE-PGC-1α mice is described in our earlier study [20]. All restriction enzymes were purchased from New England BioLabs (Ipswich, MA, USA).

Male TgSOD1^G93A^ mice (C57BL6 × SJL background) were purchased from the Jackson Laboratory (Bar Harbor, ME, USA) and bred with female actin-PGC-1α transgenic mice. The resulting litters consisted of double transgenic TgSOD1^G93A^/TgActin-PGC-1α mice, single transgenic TgSOD1^G93A^ and TgActin-PGC-1α mice, as well as wild-type (WT) mice. In our previous study [20], protection with PGC-1α over-expression was only observed in female mice (data not shown), hence we focused on female mice in this study. The mice were maintained in the transgenic mouse facility on a 12-h light–dark cycle, with free access to food and water. All animal protocols used in the study were approved by the Institutional Animal Care and Use Committee of the Icahn School of Medicine at Mount Sinai.

### 2.2. Analysis of Tissue Expression of Exogenous and Endogenous PGC-1α and Sirtuins

Four WT, NSE-PGC-1α, and actin-PGC-1α mice (two of each sex) were sacrificed by carbon dioxide inhalation. The spinal cord, brain, muscle, liver, kidney, and spleen were dissected out, frozen, and stored at −80 °C. The tissues were homogenized using a Precellys 24 (Bertin Technologies, Montigny-le-Bretonneux, France), with beads to facilitate disruption of the muscle tissue. Muscle RNA was isolated using TRIzol reagent (Invitrogen, Carlsbad, CA, USA) as per the recommended protocol from the manufacturer. RNA was isolated from the other tissues using the RNAeasy mini kit (Qiagen, Germantown, MD, USA) as per the manufacturer’s instructions and cDNA was prepared from the isolated RNAs using the Superscript III First Strand Synthesis System (Invitrogen). The hPGC-1α and mouse PGC-1α genes were amplified using DreamTaq master mix (Thermo Scientific, Waltham, MA, USA), with mouse tubulin as the housekeeping control, using the primers indicated in Table 1.

### 2.3. Assessment of Motor Performance, Body Weight, and Survival

The animals were monitored once a week for general health. Body weight was recorded starting at 9 weeks of age. Motor performance was assessed using an accelerated rotarod (7650 Ugo Basile Biological Research Apparatus, Comerio, Italy) as described earlier^18^. Briefly, the mice were placed on the rotarod, set to accelerate from 2 to 40 rotations per minute over a 5 min period. The times at which the mice could no longer hold onto the rotarod were recorded as latency to fall. Mice were acclimatized to the experimenter and the apparatus for a week prior to initiation of testing. Each mouse was tested three times per day, and the mice were tested once a week starting at age 9 weeks for the duration of the study. To keep external stimuli constant, the test was conducted in the last 4 h of the light cycle, in a room with minimal disturbances of sound, movement, light, and temperature. The end point for the survival study was determined as meeting any of the following criteria: no spontaneous breathing or movement for 1 min with no response to pain, inability to correct position in 10 s when pushed over, or total hind limb paralysis.

### 2.4. Statistics

Statistical analyses were performed using SigmaStat (version 3.0, SPSS Inc., Chicago, IL, USA). Independent measures *t*-tests were used to compare endpoints. Deterioration of motor function (rotarod testing) was assessed by the Kaplan–Meier survival analysis (Mantel–Cox log rank test) with “failure” defined as the inability of an animal to achieve at least 50% of its baseline motor performance. This endpoint was chosen because it is a measure that is distinctly related to entry criteria, is the midline of an inexorable decline in strength, and is not influenced by observer bias and animal adaptive skills used when the animal is very weak (e.g., holding on to the rotating rod without walking). A nonparametric time to failure analysis was chosen since a two-way repeated measures ANOVA could not be performed because the data failed both normality and equal-variance tests. Animals’ body weight data were analyzed by a two-way repeated measures ANOVA with Huynh–Feldt correction. ANOVA tests were followed, when significant, by the Student-Newman-Keuls multiple comparison tests. The Student-Newman-Keuls test was chosen for post hoc multiple comparisons due to its generally higher sensitivity compared to the Bonferroni test. In all tests, results with probability values less than 0.05 were considered statistically significant. Presented data are shown as mean ± SEM.

## 3. Results

### 3.1. Assessment of Tissue Transgene Expression in NSE-PGC-1α and Actin-PGC-1α Mice

**Figure 2:**
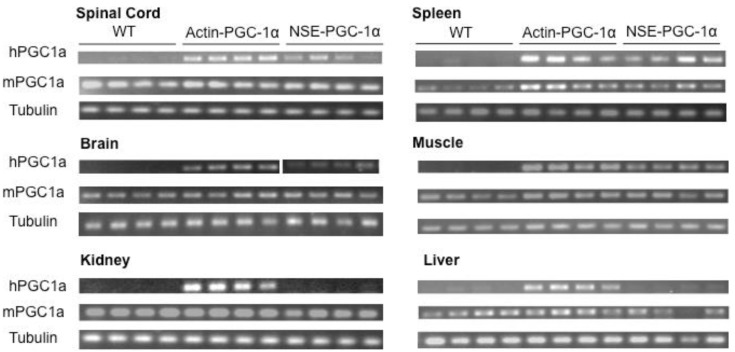
Tissue specificity of PGC-1α transgene expression. RNA was isolated from the brain, spinal cord, muscle, liver, kidney, and spleen of wild-type (WT), actin-PGC-1α, and NSE-PGC-1α mice. Expression of human PGC-1α, mouse PGC-1α, and mouse tubulin were detected following RT-PCR. Data are from *n* = 4 mice per group.

To assess the tissue-specificity of human PGC-1α expression under the NSE and actin promoters, we extracted RNA from various tissues and performed PCRs with primers specific to hPGC-1α, mouse PGC-1α, and mouse tubulin. Transgene expression was tested in spinal cord, brain, muscle, liver, kidney, and spleen. As would be expected for a transgene expressed under the actin promoter, in the actin-PGC-1α mice, the transgene was expressed in all the tissues tested (Figure 2). Whereas PGC-1α expression was present as expected in the spinal cord and brain of the NSE-PGC-1α mice and absent in the kidney and liver, there was also some expression of the transgene in the muscle and spleen (Figure 2).

### 3.2. Motor Performance, Survival, and Body Weight Were Not Affected by Actin-PGC-1α Overexpression

**Figure 3:**
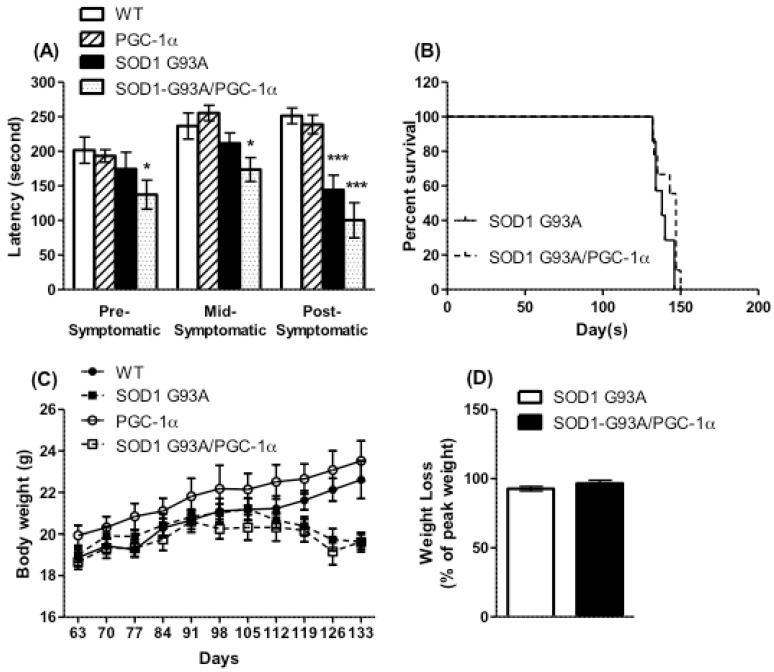
Motor function, survival, and body weight were not improved by actin-PGC-1α expression. (**A**) Motor function was assessed by the accelerating rotarod test. The double-transgenic SOD1^G93A^/actin-PGC-1α mice performed worse than the WT in pre-symptomatic (week 10), mid-symptomatic (week 14), and post-symptomatic (week 17) stages. Data are mean ± SEM from *n* = 10 WT and 11 SOD1^G93A^ mice. * *p* ≤ 0.05, *** *p* ≤ 0.001 as compared to WT by two-tailed Students’ *t*-test. (**B**) Survival was not changed in the SOD1^G93A^ mice by expression of actin-PGC-1α. (**C**,**D**) The loss of body weight seen in SOD1^G93A^ mice was not significantly altered by the expression of actin-PGC-1α.

Whole-body expression of hPGC-1α under the actin promoter did not result in significant changes in rotarod performance (Figure 3A) or body weight (Figure 3C) as compared to WT mice. Global expression of the PGC-1α transgene in SOD1^G93A^ mice did not improve the decline in motor function that set in by 14 weeks of age (Figure 3A); in fact, the double-transgenic mice performed worse than WT mice even at the pre-symptomatic stage. There was no improvement in the survival of the SOD1^G93A^ transgenic following expression of the PGC-1α transgene (Figure 3B). The SOD1^G93A^/actin-PGC-1α double transgenic mice lost weight at a rate similar to the SOD1^G93A^ transgenic starting at the 17th week of age (Figure 3C,D).

## 4. Discussion

Our previous studies supported the beneficial effects of PGC-1α expression in neurons, while the present study failed to establish beneficial effects of exogenous actin promoter-driven PGC-1α expression in multiple cells, including neurons. These results are puzzling given that actin is ubiquitously expressed. Further, we had anticipated both increased mitochondrial antioxidant capacity and suppressed inflammation, as has been observed in astrocytes [21]. Observations in line with these prior results seem to be contradictory. These results suggest that future studies involving the development of PGC-1α-targeting therapeutics focus on cell-specific lines of investigation.

The choice of ALS model could explain the differences in the protective effects of global PGC-1α expression. We found that neuron-specific, but not global, over-expression of PGC-1α was protective in the SOD1^G93A^ mouse model of ALS. Other groups found that whole-body expression of PGC-1α improved motor function and survival in SOD1^G93A DL^ mice [19], and muscle-specific PGC-1α expression in LoxSOD1^G37R^ mice improved motor function, but not survival [22]. The SOD1^G93A DL^ model has about 10 copies of the mutant *SOD1* gene and a longer life span (39–42 weeks) than the SOD1^G93A^ 1Gur/J mice (19–23 weeks) used in our study, which have higher copy numbers of the mutant *SOD1* gene (18 copies) and exhibit accelerated onset of the ALS phenotype (14–16th week onset compared to 34th week onset in the SOD1^G93A DL^ model) [23,24]. Meanwhile, the disease end stage for LoxSOD1^G37R^ mice with the highest mutation load was between 37 and 48 weeks [25].

We showed previously, in the same ALS mouse model as was used this study, that PGC-1α targeted ectopic expression under the NSE promoter significantly improves motor function and survival [20]. In the current study, systemic ectopic expression of PGC1a did not significantly improve the body weight, motor function, or survival. In our current study, we tested expression levels of PGC-1α in tissue homogenates. However, as tissue homogenates consist of multiple types of cells, drawing conclusions regarding the expression of PGC-1α in a single cell type was not feasible with our methodology. The absence of protective effects of the overexpression of PGC-1α may be a consequence of inadequate expression in cell types integral to the pathogenesis of ALS, such as motor neurons. In the current study, PGC-1α expression was higher for the actin promoter than for the NSE promoter in the homogenates of all tissues tested. It is however possible that the level of actin-PGC-1α in motor neurons is lower than levels of PGC-1α when expressed under the NSE promoter, and the actin promoter may be insufficient to induce significant beneficial effects. The reason for this discrepancy remains unknown.

PGC-1α expression is dynamic; in order to meet the tissue’s energy demands, PGC-1α is upregulated or downregulated through multiple mechanisms [26]. However, as our global expression of PGC-1α was coupled to the constitutively actively expressed β-actin promoter, there may be compensatory mechanisms occurring, by which the animal quickly adapted. Future investigations to address this possibility may better inform the discrepancy noted in symptom improvement between the global expression of PGC-1α under the β-actin promoter versus ectopic expression under the NSE promoter.

Limited information from previous studies is available regarding the relative strength of the actin versus NSE promoter in cells that are potentially relevant to the pathogenesis of ALS. For instance, in the spiral ganglion cells of mouse cochlea, EGFP levels induced by AAV transduction under the actin and the NSE promoters are similar [27]. GFP expression produced by transduction with AAV leads to approximately 3-fold higher expression under the actin versus the NSE promoter in the rat hippocampus [28]. The pertinence of this information to our study remains unclear, as these data refer to tissue homogenates rather than to neurons.

Our current study underscores the importance of characterizing the effects of a pharmacologic intervention on the specific cell type of interest that most contributes to disease pathology—in this case, motor neurons. Further studies will be dedicated to the comparative analysis of PGC-1α expression driven by actin versus NSE promoters across various cell types. Cell-specific analysis may better elucidate the relative contributions of different cell types to ALS pathology (e.g., neurons versus glia). Additionally, cell-specific analysis may indicate which cells are most relevant for predicting the effects of pharmacological agents and for monitoring the expected efficacy.

Interestingly, PGC-1α appears to have a gender-specific effect in SOD1^G93A^ mice; absence of the PGC-1α gene leads to earlier onset of the ALS phenotype only in males [29]. Female mice have previously been demonstrated to have a higher level of PGC-1α, which is subsequently reduced when ovariectomized [30]. In an additional study in ovariectomized rats, when they were treated with 17β-estradiol, PGC-1α increased in skeletal muscle [31]. We also observed a difference in protection in SOD1^G93A^ mice when over-expressing PGC-1α in neurons [20]; however, in our study, it was female mice that were protected by the higher levels of PGC-1α (data not shown). Sex hormone interaction may be driving the different responses to PGC-1α modulation.

The lack of protective effects in ALS mice when PGC-1α was globally expressed, as opposed to specifically in neurons, points to potential effects of PGC-1α expression on non-neuronal cell types such as glia. PGC-1α has been shown to regulate the transcription of myelin basic protein and cholesterol synthesis enzymes, thereby affecting myelination in the brain [32]. Pioglitazone, an anti-diabetic drug and agonist of peroxisome proliferator-activated receptor-γ (PPARγ), improved motor function and survival in SOD1^G93A^ mice by protecting neurons and reducing the activation of microglia and astrocytes in spinal cord [33,34]. Another study indicated that neuronal protection by pioglitazone was mediated through neuronal p38 mitogen-activated protein kinase and reduced glial activation via nuclear factor-κB, potentially independent of PPAR-γ [35]. Our previous study showed that neuron-specific expression of PGC-1α was capable of reducing the activation of p38 mitogen-activated protein kinase [20], indicating a mechanism of action similar to pioglitazone in neuronal protection. However, it is not clear whether the interaction of PGC-1α with PPAR-γ would have a similar action on glial proliferation to pioglitazone. Of note, a clinical trial with pioglitazone did not show additional improvement in patients with ALS who were on riluzole treatment [36]. This further underlines the importance of understanding the pathways by which PGC-1α exerts its effects on neuronal and whole-body function in ALS, and of validating the beneficial effects of modulating neuron-specific expression of the co-activator using other models of ALS.

## 5. Conclusions

Our study demonstrated that while the over-expression of PGC-1α in neurons in an ALS mouse model has protective effects, overexpression throughout the body driven by the actin promoter does not confer protective effects. Further investigations are underway to determine the exact mechanisms for the difference in phenotypic response to differential expression of PGC-1α. These investigations will continue to compare PGC-1α expression, induced by multiple promoters, and will possibly target independent cell types in the brain and periphery. Further, the main information provided by the study is that the expression of PGC-1α throughout the body is insufficient to induce beneficial effects. The study suggests that it is essential to achieve a level of PGC-1α above a certain threshold in order to confer protective effects. This study suggests for the first time that PGC-1α expression in cell types other than neurons, thought to be important for the ALS pathology (e.g., glial cells), cannot substitute for robust neuronal expression. This evidence has profound implications for future investigations exploring novel therapeutics in ALS targeting, for example, multiple mechanisms in independent cell types in the brain.

## Figures and Tables

**Table 1:** PCR primers used in the study.

Gene	Primer Orientation	Sequence (5′–3′)
***Genotyping Primers***		
pCAGGS Human PGC-1α	Forward Reverse	GGTTCGGCTTCTGGCGTG CCACAGGGAGACTGTCTAGTGTC
Mouse IL-2	Forward Reverse	CTAGGCCACAGAATTGAAAGATCT GTAGGTGGAAATTCTAGCATCATCC
***qRT-PCR Primers***		
Human PGC-1α	Forward Reverse	CAGGCAGTAGATCCTCTTCAAG TCCTCGTAGCTGTCATACCTG
Mouse PGC-1α	Forward Reverse	AAGGTCCCCAGGCAGTAGAT CATAGCTGTCGTACCTGGGC
Mouse tubulin	Forward Reverse	TAGCAGAGATCACCAATGCC GGCAGCAAGCCATGTATTTA

## Abbreviations

ALS	amyotrophic lateral sclerosis,
fALS	familial ALS,
sALS	sporadic ALS,
SOD1	superoxide dismutase 1,
PGC-1α	peroxisome proliferator-activated receptor γ coactivator-1 α,
PPAR-γ	peroxisome proliferator-activated receptor γ,
NSE	neuron-specific enolase,
